# Cardiovascular and renal diseases in type 2 diabetes patients: 5-year cumulative incidence of the first occurred manifestation and hospitalization cost: a cohort within the French SNDS nationwide claims database

**DOI:** 10.1186/s12933-023-02101-1

**Published:** 2024-01-09

**Authors:** Patrick Blin, Michael Joubert, Patrick Jourdain, Philippe Zaoui, Estelle Guiard, Dunia Sakr, Caroline Dureau-Pournin, Marie-Agnès Bernard, Régis Lassalle, Florence Thomas-Delecourt, Sébastien Bineau, Nicholas Moore, Cécile Droz-Perroteau

**Affiliations:** 1 Univ. Bordeaux, INSERM CIC-P 1401, Bordeaux PharmacoEpi, 146 rue Léo Saignat, Bordeaux, 33000, 33076 France; 2 Diabetes Care Unit, Caen University Hospital, UNICAEN, Caen, 14033 France; 3 APHP Bicêtre University Hospital, Bicêtre, France; 4 Grenoble University Hospital, Grenoble, France; 5 Astra-Zeneca, Courbevoie, France

**Keywords:** Type 2 diabetes, Myocardial infarction, Stroke, Peripheral arterial disease, Heart failure, Chronic kidney disease, Cardiorenal disease, Mortality, Incidence, Claims database

## Abstract

**Background:**

Myocardial infarction (MI), stroke, peripheral arterial disease (PAD), heart failure (HF) and chronic kidney disease (CKD) are common cardiovascular renal diseases (CVRD) manifestations for type 2 diabetes. The objective was to estimate the incidence of the first occurring CVRD manifestation and cumulative hospitalization costs of each CVRD manifestation for type 2 diabetes without CVRD history.

**Methods:**

A cohort study of all type 2 diabetes free of CVRD as of January 1st 2014, was identified and followed-up for 5 years within the French SNDS nationwide claims database. The cumulative incidence of the first occurring CVRD manifestation was estimated using the cumulative incidence function, with death as a competing risk. Cumulative hospitalization costs of each CVRD manifestations were estimated from the perspective of all payers.

**Results:**

From 2,079,089 type 2 diabetes without cancer or transplantation, 76.5% were free of CVRD at baseline with a mean age of 65 years, 52% of women and 7% with microvascular complications history. The cumulative incidence of a first CVRD manifestation was 15.3% after 5 years of follow-up with a constant linear increase over time for all CVRD manifestations: The most frequent was CKD representing 40.6% of first occurred CVRD manifestation, followed by HF (23.0%), then PAD (13.5%), stroke (13.2%) and MI (9.7%). HF and CKD together reached about one patient out of ten after 5 years and represented 63.6% of first CVRD manifestations. The 5-year global cost of all CVRD hospitalizations was 3.9 billion euros (B€), i.e. 2,450€ per patient of the whole cohort, with an exponential increase over time for each specific CVRD manifestation. The costliest was CKD (2.0 B€), followed by HF (1.2 B€), then PAD (0.7 B€), stroke (0.6 B€) and MI (0.3 B€).

**Conclusions/interpretation:**

While MI, stroke and PAD remain classic major risks of complications for CVRD-free type 2 diabetes, HF and CKD nowadays represent individually a higher risk and cost than each of these classic manifestations, and jointly represents a risk and a cost twice as high as these three classic manifestations all together. This should encourage the development of specific HF and CKD preventive strategies.

## Study registration

European database of post-approval studies (www.Encepp.eu) as EUPAS37569.

## Introduction

More than half a billion of the world’s population aged 20 to 79 is affected by diabetes, mostly type 2 diabetes. with 6.7 million deaths and nearly one trillion US dollars in health expenditure per year [1, 2]. Diabetes is a leading cause of cardiovascular diseases, classically myocardial infarction (MI), stroke, peripheral arterial disease (PAD) for macroangiopathy, and diabetic nephropathy, neuropathy and retinopathy for microangiopathy, major causes of disability and death [3–9]. The development of sodium-glucose co-transporter-2 inhibitors (SGLT2i) and results from their randomized control trials, as well as some recent epidemiologic studies also highlighted the importance of the risk of heart failure (HF), chronic kidney disease (CKD) and of their association as cardiorenal diseases (CRD) in patients with type 2 diabetes [10–19]. A recent population-based cohort study demonstrated that four out of five persons with type 2 diabetes will suffer from a major cardiovascular or renal disease (CVRD) over their lifetime [20].

However, the risk of the first occurring CVRD manifestation is not well known. A large multinational cohort study was designed with a core protocol to estimate this risk for patients initially free of CVRD, as well as the impact of each CVRD manifestation on health care utilization through the cumulative cost of CVRD hospitalization over time, using various databases (primary care, pharmacist, hospital, claims, registry database…) in different countries. First results of the risk were published for six countries, three from a sample of the population using primary and hospital care (England, Germany, Netherlands), one from a sample of the population using only hospital information (Japan) and two from the whole population of two Nordic countries using only hospital information (Norway, Sweden) [17]. Hospitalization costs were published later for six countries, four from a sample of the population using primary and hospital care (Canada, Italy, Portugal, Spain), one from a sample of the population using hospital care (Japan), and one from the whole population of a Nordic countriy using only hospital information (Sweden) [21]. Finally, both incidence and cost criteria were estimated only in Sweden, a relatively small country and using only hospital information.

## Methods

### Aim, design and setting of the study

The aim of the study was to estimate the 5-year cumulative incidence of the first occurred CVRD manifestation and the 5-year cumulative hospitalization cost of each CVRD manifestation for all type 2 diabetes free of CVRD at baseline from the whole population of a large European country, according to primary and hospital care.

A 5-year follow-up cohort study was designed within the French SNDS nationwide claims database with 4 years of database history before patient inclusion. The SNDS links the national mandatory public health insurance system claims database to the national hospital-discharge summaries database and the national death registry, using a unique national pseudonymised identifier [22, 23]. It currently includes about 99% of the French population, more than 67 million persons from birth (or immigration) to death (or emigration), even if a subject changes occupation or retires, and irrespective of socioeconomic status. It contains all reimbursed outpatient healthcare expenditures, public and private hospital-discharge summaries with International Classification of Diseases 10th revision (ICD-10) discharge diagnoses, and long-term disease registration (with ICD-10 codes) allowing 100% reimbursement for expenditure related to this long-term disease. Date of death is available and causes of death are uploaded gradually but were not available at the time of data of extraction for this study.

### Characteristics of participants

All type 2 diabetes adult patients free of CVRD identified on January 1st, 2014 (index date) with a 4-year database history and 5-years of follow-up (or until death) were included in the cohort after exclusion of those with a cancer history, organ transplantation or immunosuppressive treatment for transplantation. The end of the study follow-up (2018) comes before the SGLT2i reimbursement in France and hence their use in this country. Inclusion and exclusion criteria were defined using the 4-year database history information and long-term disease registrations before the index date. Type 2 diabetes was defined as patients with type 2 diabetes hospital discharged or long-term disease registration diagnosis (E11 ICD-10 code), or with ≥ 3 non-insulin antidiabetic drugs dispensing (or ≥ 2 if at least one big packaging) [9, 24]. CVRD-free patients were defined as patients without hospital discharge or long-term disease registration diagnosis of myocardial infarction (I21, I22, I25.2, I25.6 ICD-10 codes), angina or unstable angina (I20, I25.1, I25.5 ICD-10 codes), coronary revascularization, nitrate dispensing, atrial fibrillation (AF) (I48 ICD-10 codes), HF (I50, I11.0, I13.0, I13.2 ICD-10 codes), peripheral artery disease (PAD) (I70.2, I73, I74.2-9 ICD-10 codes), peripheral artery revascularization, stroke or transient ischemic attack (I60-I66, I69 and G45 ICD-10 codes), CKD (N17-N19, I120, I129, I131, I139, E102, E112, E122, E132, E142, N083 ICD-10 codes) or dialysis before the index date.

### Outcomes

The first occurred CVRD manifestation was defined as first hospitalization during the follow-up with main or associated discharge diagnosis of MI (I21-I22 ICD-10 codes), or stroke (I60-I63 ICD-10 codes), or PAD (I70.2, I73, I74.2-9 ICD-10 codes), or HF (I50, I11.0, I13.0, I13.2 ICD-10 codes), or CKD (N17-N19, I120, I129, I131, I139, E102, E112, E122, E132, E142, N083 ICD-10 codes), codes validated and used in several previous published studies [9, 25–32]. CRD was defined as occurrence of HF or CKD. If more than one CVRD event occurred on the same date, the first occurred manifestation was defined in the following order of preference: firstly, main diagnosis, then the acute event (MI or stroke), then a diagnosis of CVRD during an intensive care unit stay, and then randomly selected giving equal probabilities to the CVRD manifestation presented. A sensitivity analysis was performed using only main diagnosis for outcome definitions. In order to estimate the relative impact of each CVRD manifestation on health care utilization over time, cumulative hospitalization cost of each outcome took into account first and repeated CVRD hospitalizations during the follow-up, including rehabilitation centres, using main or associated discharge diagnosis and same ICD10-codes.

### Statistical analysis

Patient’s characteristics were presented for the CVRD free population and the CVRD comorbidity population and compared using t-test for continuous variables and Chi-square test for binomial and categorial variables. For each outcome, patients were right censored after the first occurrence of the outcome, lost to follow-up, death, or end of the study, whichever came first. Incidence rate was estimated by the number of the first corresponding occurred outcome divided by the number of person-years (PY) of follow-up for this outcome. The 5-year cumulative incidence of each CVRD manifestation was estimated for the overall disease-free type 2 diabetes population and according to three age classes (< 65, 65–74, ≥ 75 years old) using cumulative incidence function taking into account death as a competing risk. The 5-year cumulative hospitalization cost was estimated for each CVRD manifestation over time from the all-payers perspective. Statistical analyses were conducted by the Bordeaux PharmacoEpi using SAS® (SAS Institute, latest current version, North Carolina, USA).

## Results

**Fig. 1:**
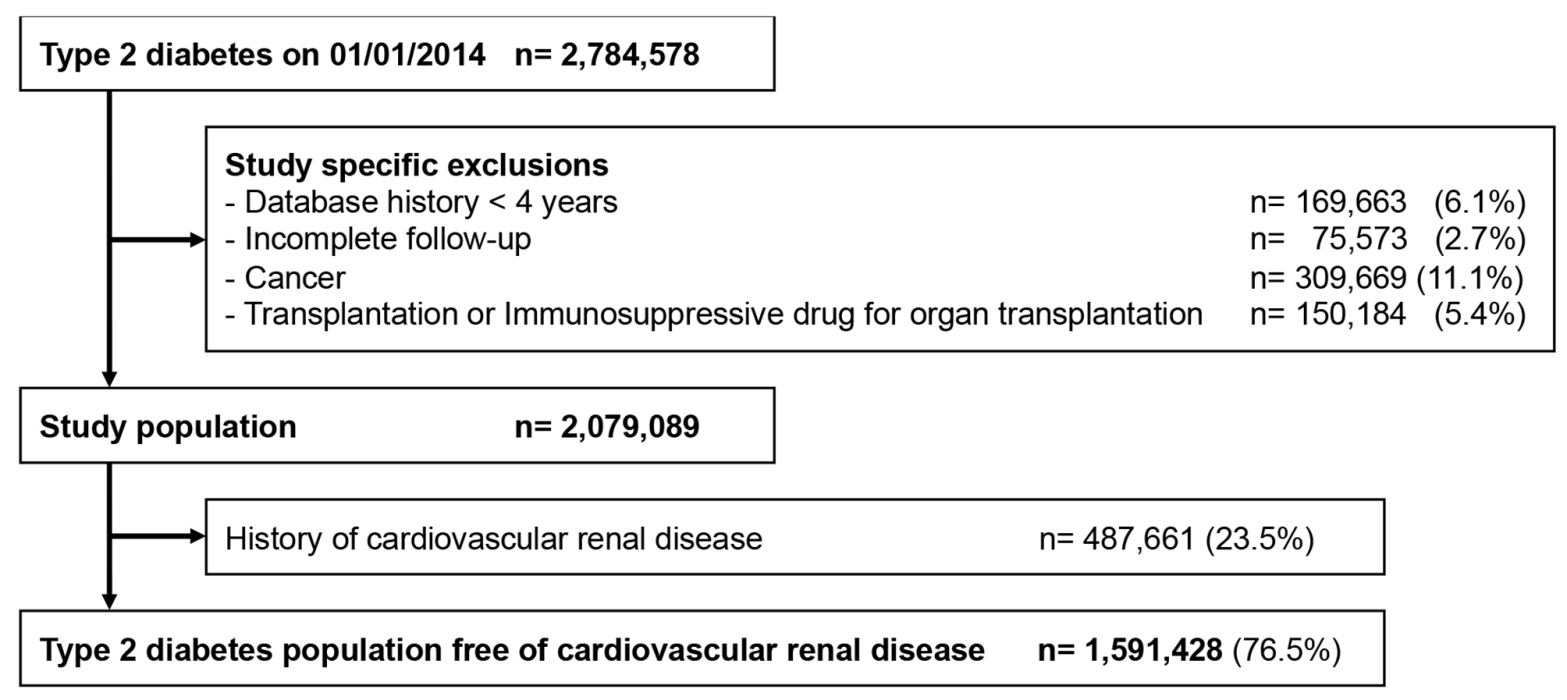
Flow chart of the type 2 diabetes population free of cardiovascular renal disease

From 2,079,089 type 2 diabetes adults with a 4-year database history, no cancer or transplantation, and full study follow-up, around three-quarters (76.5%) were free of CVRD at baseline (Fig. 1). The mean age of the CVRD-free population was 65 years, with a sex-ratio close to one (48.2% of men), very few having microvascular complications, mostly treated with antidiabetic monotherapy, sulfonylurea being the most used antidiabetic and 10,4% using insulin, while 3 out of 4 having a cardiovascular drug dispensing, for treatment or prevention, within the 3 months before the index date (Table 1). The picture is completely different to that of patients with at least one CVRD comorbidity (Table 1).

**Table 1:** type 2 diabetes characteristic at inclusion

	CVRD free n = 1,591,428	CVRD Comorbidity n = 487,661	*p*
**Follow-up (years)**, mean ± (sd)	4.8 (0.7)	4.4 (1.3)	< 0.001
**Age (years)**, mean ± (sd)	65.2 (12.1)	72.0 (11.5)	< 0.001
**Male**, %	48.2	55.7	< 0.001
**4-year database and LTD history**, %			
Diabetic eye complications	1.9	7.3	< 0.001
Diabetic neuropathy	1.4	8.6	< 0.001
Severe hypoglycaemia	0.6	2.7	< 0.001
Keto-lactate acidosis	0.5	2.1	< 0.001
Lower limb amputations	0.1	1.0	< 0.001
Myocardial Infarction	-	11.0	
Stroke	-	17.5	
Heart failure	-	16.8	
Peripheral arterial disease	-	14.4	
Chronic kidney disease		22.7	
Cardio-vascular and renal disease	-	4.8	
Chronic obstructive pulmonary disease	1.2	6.1	< 0.001
Major Bleeding	0.6	4.5	< 0.001
**Within 3 months before the index date**			
**Last antidiabetic treatment dispensing, %**			< 0.001
None	13.3	13.1	
Monotherapy	42.5	39.1	
Bitherapy	22.2	18.4	
Tritherapy or more	11.5	8.6	
Insulin	10.4	20.8	
**Last antidiabetic drug dispensing, %**			
Metformin	52.9	45.7	< 0.001
Sulfonylurea	29.1	26.1	< 0.001
DPP-4 inhibitors	11.7	11.2	< 0.001
Ascarbose	3.2	2.9	< 0.001
Metiglinides	5.8	9.0	< 0.001
GLP1-RA	3.0	3.7	< 0.001
Insulin	10.4	20.8	< 0.001
**Cardiovascular drug dispensing, %**			
Low dose aspirin	22.2	41.2	< 0.001
Statins	40.1	55.5	< 0.001
Antihypertensives	5.1	8.5	< 0.001
ACEI or ARB	53.7	68.9	< 0.001
Beta blockers	23.8	46.9	< 0.001
Calcium channel blocker	17.5	30.1	< 0.001
Low ceiling diuretics	1.1	1.9	< 0.001
P2Y_12_ antagonists, %	3.3	20.0	< 0.001

ACEI: angiotensin-converting enzyme inhibitors, ARB: angiotensin receptor blockers, CVRD: cardiovascular renal disease; LTD: long-term disease registration, sd: standard deviation

**Fig. 2:**
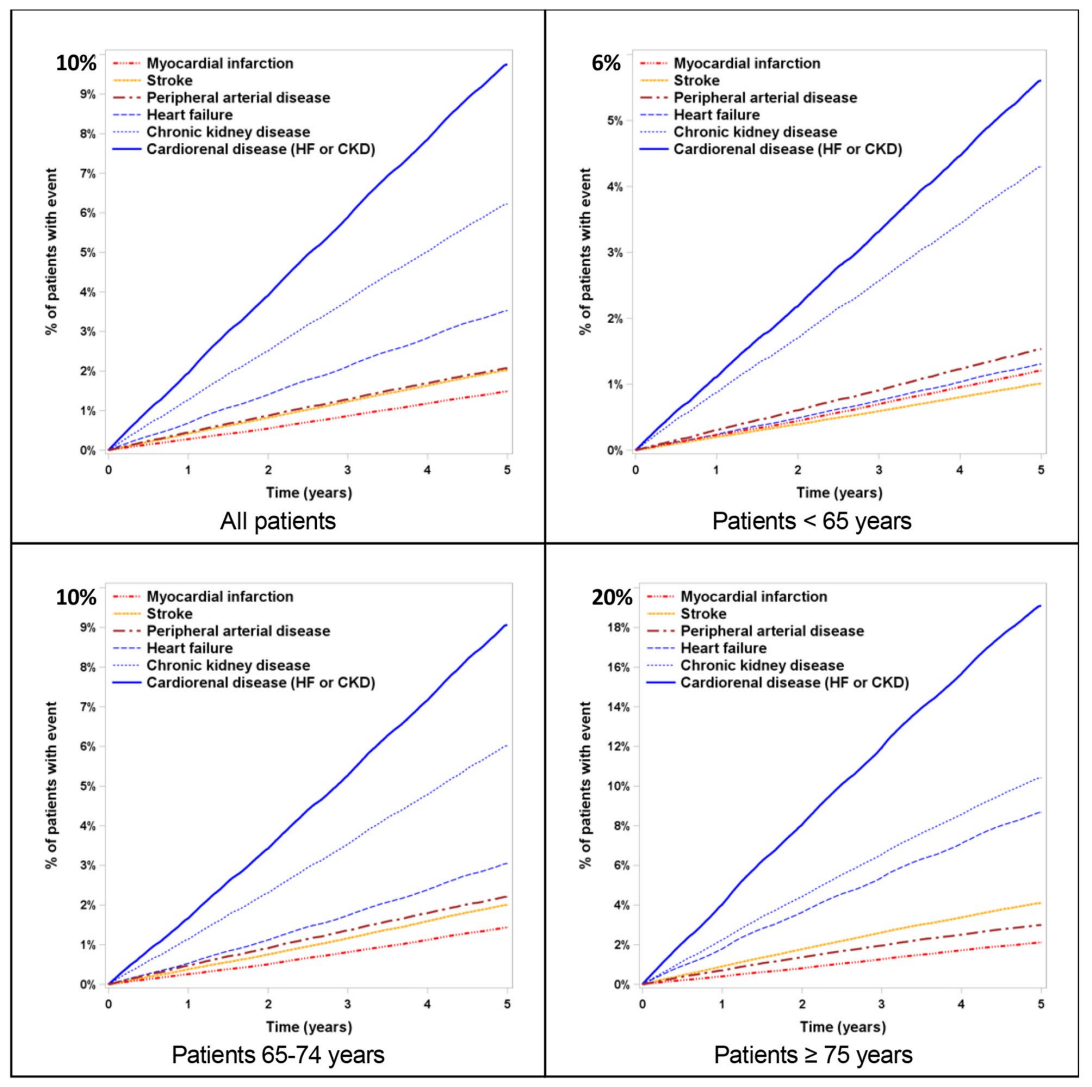
5-year cumulative incidence of the first occurred cardiovascular renal disease manifestation for type 2 diabetes free of cardiovascular renal disease at baseline

The outcome incidence rate for the CVRD-free cohort was 3.9 per 1,000 person-years (‰PY) for MI, 5.1‰PY for stroke, 6.2‰PY for PAD, 11.0‰PY for HF, 17.9‰PY for CKD, and 24.5‰PY for CRD (HF and/or CKD) with 17.8‰PY of deaths. The cumulative incidence of a first CVRD manifestation was 15.3% after 5 years of follow-up with a constant linear increase over time for all manifestations (Figs. 2), 1.5% of the patients for MI, 2.0% for stroke, 2.1% for PAD, 3.5% for HF, 6.2% for CKD; CRD reaching nearly one patient out of ten (9.8%). CKD was therefore the most frequent manifestation and represented 40.6% of the first occurred CVRD manifestation, followed by HF 23.0%, and together with CRD 63.6%, far ahead of MI (10%), stroke (13%) and PAD (13%).

The cumulative incidence increased with age, and similar proportion of CVRD manifestations were observed whatever the age-group, except for HF which represented 14% of the manifestations before 65 to reach 31% for those aged 75 or older (Fig. 2). The sensitivity analysis using only main diagnosis for outcome definition showed the same trend with a 5-year overall cumulative incidence of a first CVRD manifestation at 8.8%: 1.5% for MI, 2.0% for stroke, 1.1% for PAD, 2.3% for HF, and 1.9% for CKD. With this restrictive definition, CRD still represented about half (47.7%) of the first occurred CVRD manifestation as main diagnosis for hospitalization.

**Fig. 3:**
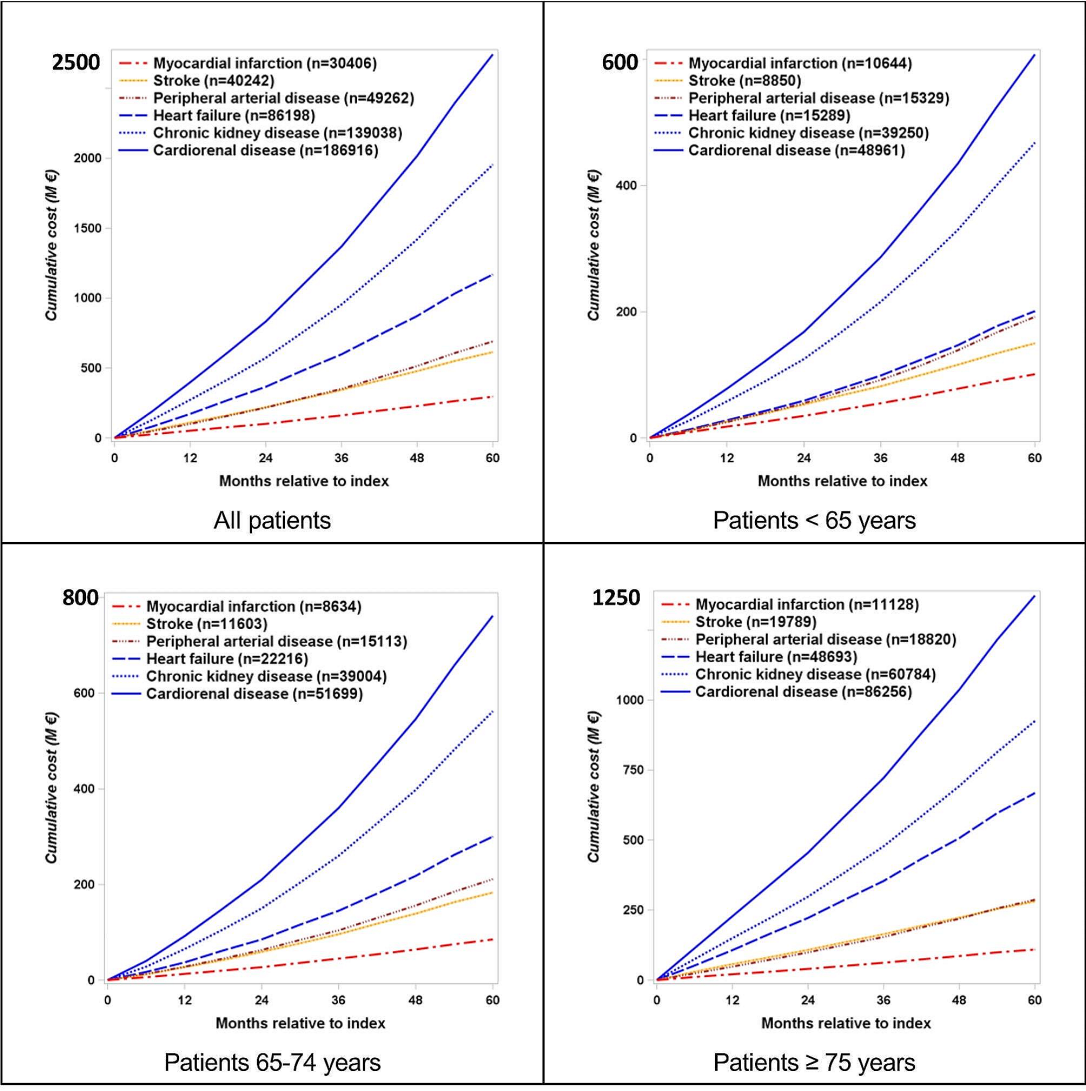
5-year cumulative hospital costs of each cardiovascular renal disease manifestation (first and repeated events) for type 2 diabetes free of cardiovascular renal disease at baseline

The 5-year global cost of all CVRD hospitalizations, including the first and repeated events, was 3.9 billion euros (B€), i.e., 2,450€ per patient, with an increase weakly exponential over time for each specific CVRD manifestation (Fig. 3). The 5-year cumulative hospital costs were 0.3 B€ for MI, 0.6 B€ for stroke, 0.7 B€ for PAD, 1.2 B€ for HF and 2.0 B€ for CKD. It was 2.7 B€ for CRD hospitalizations (CKD and HF together) compared to 1.5 B€ for MI, stroke and PAD together. Sum of the hospital costs for each specific CVRD was 22% higher than the overall cost because some hospitalizations may have involved two or more CVRD diagnoses when considering primary and associated diagnoses. The 5-year global cost of all CVRD hospitalizations for the three age-groups showed a clear increase of HF and CKD hospitalization costs according to age, while the increase is relatively weak for MI, stroke and PAD.

## Discussion

This nationwide cohort study from a large country shows that three quarters of 2 million adult type 2 diabetes without cancer or transplantation history were free of CVRD at baseline, and that 15.3% of them had a first CVRD manifestation after five years of follow-up, with a constant linear increase over time. The most frequent of first occurred CVRD manifestations was CKD (40.6%) followed by HF (23.0%) which were respectively about four and two times more frequent than MI (10%), stroke (13%) and PAD (13%). CRD (HF and/or CKD) represented almost two-thirds (63.6%) of the first occurred CVRD manifestation. In parallel, the global cumulative cost of CVRD hospitalizations reached nearly 4 billion euros after a 5-year follow-up period for these patients free of CVRD at baseline, and 2,450€ per patient of the cohort. The costliest CVRD manifestation was CKD followed by HF, respectively 6.6 and 4.0 times more costly than for MI, and respectively about three and two times more costly than stroke and PAD. The cost of hospitalization with CRD diagnosis represents more than two thirds (69.2%) of all CVRD hospitalizations. It is important to remind that the end of the study follow-up precedes the reimbursement of SGLT2i in France and therefore the results presented here are not influenced by the use of this therapeutic class. It is also important to note that the study ended shortly before the 2020-21 COVID-19 lockdowns and their impact on care management, which most likely would have had a major impact on the study results.

The proportion of type 2 diabetes free of CVRD at baseline was higher in this SNDS study than the two thirds found in a previous 6-country study using the same core protocol [17]. This may correspond to the well-known north-south cardiovascular risk demonstrated by the Monica project [33], with higher incidence in northern European countries such as Norway, Sweden, England, and to a lesser extent Germany and Netherlands, compared to France. This was also due to the information available in the various databases. Indeed, three of the six countries (Norway, Sweden and Japan) only took into account inpatients who were generally more severe than the rest of the population with healthier outpatients. Furthermore, the proportion of patients free of CVRD at baseline in our study could have been underestimated with patients having CVRD history before the 4-year history taking into account the patient selection, and without long-term disease registration for the corresponding CVRD history. However, the general characteristics of the SNDS population free of CVRD at baseline were really close to those of the previous 6-country study, with exactly the same mean age, a few more women, similar distribution of antidiabetics, except two less use of insulin, and similar use of cardiovascular prevention drugs. The prevalence of CVRD complications was also higher in the CAPTURE study which included 9,823 adults with type 2 diabetes across 13 countries from five continents, showing a CVD prevalence of 34.8% (95% confidence interval [CI] 32.7–36.8) [34]. This higher CVD burden in the CAPTURE study could be explained by a recruitment bias as more than half of the population study was provided by specialist care centers, probably managing more severe patients.

The incidence rates of the SNDS study were somewhat different from the overall results of the 6-country study: close for CKD (17.9 vs. 18.5‰PY) and about 2.5‰PY less for HF, stroke and MI (11.0 vs. 13.4, 5.1 vs. 7.5, and 3.9 vs. 6.5 respectively), and slightly higher for PAD (6.2 vs. 5.0‰PY). However, incidence rates of outcomes present variations according to the country management and the type of database. These variations can be partly explained by the north-south gradient of cardiovascular disease [33]. This should be the case for Japan, which has one of the lowest cardiovascular risks among industrialized countries [35], while it had one of the higher CRD incidence rates in the 6-country study after 2.5 years of follow-up, probably because only CVRD-free inpatients were taken into account in their study. Conversely, the lowest incidence rates were observed in England with the GP CPRD electronic medical record which probably missed hospitalizations for a number of patients. Our incidence rates were close to those of Germany which use similar claims database as the French SNDS. Nevertheless, the global picture with respective share of cumulative incidences of a first CVRD manifestation was similar across the 6 countries, as well as in France, with CKD as the first CVRD manifestation followed by HF, which together accounted for 63% of the first CVRD event in both studies. A large type 2 diabetes cohort from the national Swedish registry and followed-up for 5.7 years showed that type 2 diabetes without other risk factors had no or weak increase risk of MI, stroke or mortality compared to the general population, while they had a clear increase of HF risk [12]. This important HF incidence is particularly worrying in view of the very poor prognosis of this pathology with a reduced life expectancy compared to myocardial infarction and even to most solid cancers (except lung) [36]. It has also been shown that the prognosis of heart failure is even worse in association with diabetes, with a factor 2 higher all-cause mortality in this population [37].

It was about the same global picture of cumulative costs of CVRD hospitalizations, including first and repeated events, compared to the second 6-country study [21], with the same distribution of costs between the different CVRD, about 60% of cost related to CRD and a cost per patient in the same range for the three countries with at least a 5-year of follow-up (Canada, Spain and Sweden). A predictive model for estimating the cost of incident diabetes complications showed similar results, with the three most costly complications being, in descending order, heart failure, end-stage renal disease and gangrene (annual expected cost for 10 000 adults with diabetes: $ 7 320 287; $ 4 225 384; $ 2 844 381, respectively) [38].

The burden of HF and CKD in patients with type 2 diabetes. as well as the cost associated with it, encourages the consideration of early detection measures as well as preventive therapeutic strategies to limit the impact of these two complications. According to the ENTRED study on annual screening for CKD in France, annual albuminuria testing is performed in less than 30% of patients with diabetes, opening up prospects for major improvements in this screening [39]. Beyond screening for complications, our data raised the question of the benefit of preventive use of specific treatments that could reduce the incidence of new cases of HF and CKD. A recent study, performed on a large international database, explored the incidence of new onset HF in patients with diabetes, depending on whether or not they were treated with SGLT2-inhibitors. After matching these two populations by propensity score, the authors showed that the risk of developing heart failure, for patients who were initially free of it, was reduced by 30% in the group receiving SGLT2-inhibitors compared to the control group [40]. This protective effect was not explored in our study, for which our method was not designed to answer such a question, and also too few patients exposed at index date (Table 1).

The main limitation of our study is to be based on administrative data with diagnostic coding related to the hospital billing system that could influence the choice of diagnosis. However, this coding system is regularly checked and audited by French health authorities with a very good positive predictive value and excellent negative positive value for diagnoses studied here: respectively 88.6% and 94.8% for MI, 71.3% and 97.0% for cerebral vascular disease, 84.8% and 98.6% for PAD, 88.6% and 94.8% for HF and 91.1% and 98.0% for CKD [41]. Furthermore, in a previous publication, cerebral vascular disease also included transient ischemic accidents, while only stroke was taken into account in this SNDS study. Another SNDS publication showed a positive predictive value at 88.3% for stroke as the main diagnosis from a random sample of 31 hospitals [32]. On the other hand, the use of such claim database is also a strength insofar as all health events are listed, opening up the prospect of analyzing other events, such as cancers or neurodegenerative pathologies, in subsequent studies.

Conversely, the study did not take into account outpatient diagnosis. For acute events requiring hospitalization, such as MI and stroke it should not change much, but it underestimates low-severity stages of chronic CVRD such as PAD, HF and CKD. Indeed, we can expect that patients with non-severe PAD, HF and/or CKD, or in the early days of the PAD, HF and/or CKD were not hospitalized.

This is pretty well shown with the sensitivity using only main diagnosis for outcome definition. Compared to main analysis using main and associated diagnoses, it showed exactly the same 5-year cumulative incidence for stroke and MI, a third less for HF, about half less for PAD, and 70% less for CKD; reflecting the fact that stroke and MI are the main causes of the hospitalization for quite all patients, while HF, PAD and above all CKD are more often a comorbidity discovered or reported during a hospitalization for another reason. Nevertheless, with a high specific definition using main diagnosis hospitalization, HF and CKD jointly still represented about half of the first occurred CVRD manifestations with main diagnosis for hospitalization.

In conclusion, while MI, stroke and PAD remain classic major first occurred CVRD manifestations for people with type 2 diabetes, HF and CKD nowadays represent individually a higher risk and cost than each of these classic manifestations, and jointly represent a risk and a cost twice as high as these three classic manifestations all together. This should encourage the development of early recognition markers and specific preventive strategies.

## Data Availability

The dataset analysed during the study are not publicly available due to the French law which forbid to share individual data from a SNDS data extraction. However, any researcher from a European entity can submit a file to the Health Data Hub (https://www.health-data-hub.fr) to have access to the same SNDS data extraction.

## Abbreviations

B€: Billion Euros
CI: confidence interval
CKD: chronic kidney disease
CRD: cardiorenal disease
CVD: cardiovascular disease
CVRD: cardiovascular renal disease
HF: heart failure
ICD-10: International Classification of Diseases 10th revision
MI: myocardial infarction
PAD: peripheral arterial disease
SGLT2i: sodium-glucose co-transporter-2 inhibitors
